# Molecular iodine-promoted oxidative cyclization for the synthesis of 1,3,4-thiadiazole-fused- [1,2,4]-thiadiazole incorporating 1,4-benzodioxine moiety as potent inhibitors of α-amylase and α-glucosidase: *In vitro* and *in silico* study

**DOI:** 10.3389/fchem.2022.1023316

**Published:** 2022-10-06

**Authors:** Rafaqat Hussain, Mazloom Shah, Shahid Iqbal, Wajid Rehman, Shoaib Khan, Liaqat Rasheed, Haseena Naz, Hanan A. Al-ghulikah, Eslam B. Elkaeed, Rami Adel Pashameah, Eman Alzahrani, Abd-ElAziem Farouk

**Affiliations:** ^1^ Department of Chemistry, Hazara University, Mansehra, Khyber Pakhtunkhwa, Pakistan; ^2^ Department of Chemistry, Abbottabad University of Science and Technology (AUST), Abbottabad, Pakistan; ^3^ Department of Chemistry, School of Natural Sciences (SNS), National University of Science and Technology (NUST), Islamabad, Pakistan; ^4^ Department of Chemistry, College of Science, Princess Nourah bint Abdulrahman University, Riyadh, Saudi Arabia; ^5^ Department of Pharmaceutical Sciences, College of Pharmacy, AlMaarefa University, Riyadh, Saudi Arabia; ^6^ Department of Chemistry, Faculty of Applied Science, Umm Al-Qura University, Makkah, Saudi Arabia; ^7^ Department of Chemistry, College of Science, Taif University, Taif, Saudi Arabia; ^8^ Department of Biotechnology College of Science, Taif University, Taif, Saudi Arabia

**Keywords:** synthesis, 4-benzodioxin, thiadiazole-fused-[1,4]-thiadiazole, α-amylase, α-glucosidase, molecular docking

## Abstract

Twenty-five analogs were synthesized based on 1,3,4-thiadiazole-fused-[1,2,4]-thiadiazole incorporating 1,4-benzodioxine moiety **(1–25)** and then tested for the antidiabetic profile. The entire afforded derivatives showed varied inhibition profiles ranging between 0.70 ± 0.01 and 30.80 ± 0.80 μM (against α-amylase) in comparison to standard acarbose (12.80 ± 0.10 μM). Similarly, synthetics analogs also displayed a varied range of α-glucosidase activity ranging from 0.80 ± 0.01 μM to IC_50_ = 29.70 ± 0.40 μM (against α-glucosidase) as compared to standard acarbose (IC_50_ = 12.90 ± 0.10 μM). Among synthesized analogs, compound **22** showed excellent potency due to the presence of di-hydroxy substitutions at the 2,3-position of the aryl ring. For all analogs, the structure–activity relationship was carried out based on the pattern of substitutions around the aryl ring, and further, the potent analogs were subjected to a molecular docking study to analyze how active residues of targeted enzymes interact with active parts of newly prepared analogs. The result obtained shows that these compounds furnish several key interactions with enzyme active sites and, hence, enhanced their enzymatic activities.

## 1 Introduction

A high concentration of glucose in plasma results in diabetes, which is a major metabolic problem (Mitrakou et al., 1992). Diabetes, which occurs in two types, namely, type-1 and type-2, is one of the primary problems. Inadequate insulin production is a defining feature of type-2 diabetes, commonly referred to as non-insulin-subordinate diabetes (Fall et al., 2006). Because type-2 diabetes is regarded as a preventable illness, it is more significant than type-1 diabetes. An imbalance between glucose consumption and insulin release is the cause of type-2 diabetes. The fundamental strategy for preventing type-2 diabetes is blood sugar control (Baron, 1998). Through advice and food management, it is reasonable to achieve this target with active insulin release (Goldberg, 1998; Kahn, 2001; Rabasa‐Lhoret and Chiasson, 2003). It was suggested by the United Kingdom Prospective Diabetes Study (UKPDS) that one of the major treatments for diabetes is diet control. Eating practice that depends on the blood sugar list of foodstuff is presently one of the largely suggested health remedies. It was concluded that nutritional treatment can be used at the same time as former clinical medicines to achieve a greater impact (Jenkins et al., 1981; Wolever et al., 1994; American College of Sports Medicine, 2000). In some cases, this is the inconvenience of restricting the types and quantity of food consumed. A further probable arrangement is to reduce the speed of glucose absorbance from the small digestive area by holding up the processing of nutritional starch, the important nutritional concern of glucose (Rabasa‐Lhoret and Chiasson, 2003). For practical considerations, accommodations, and avoidance of outcomes, this strategy is seen as being more effective than managing insulin discharge (Kahn, 2001). The disruption of enzymes that convert dietary starch into glucose, such as α-amylase and α-glucosidase, has received attention as a method to control blood sugar levels (Ahrén et al., 2004; Ali et al., 2006; Geng et al., 2008). α-Amylase catalyzes the hydrolysis of (1,4)-glycosidic bonds, releasing glucose and maltose from starch (Takkinen et al., 1991; Svensson and Søgaard, 1993), while α-glucosidase releases glucose from maltose or possibly sucrose (Anderson et al., 2003; Mohan et al., 2007). The two of them are discharged in the little digestive tract, while just α-amylase is found inside saliva. The retention of glucose in the circulatory system can be delayed to lower type-2 diabetes by reducing these two proteins (α-amylase and α-glucosidase). These efforts have been undertaken to differentiate between inhibitors of α-glucosidase and α-amylase that can be used as food or food additives. However, the evidence (Seo et al., 2005; Wakita et al., 2005) demonstrated that several sugar-like phenolic substances have the potential to block α-glucosidase. The majority of research on α-glucosidase and α-amylase inhibitors has been concentrated on phenolic substances (Hermeking, 2007; Bhandari et al., 2008).

Blood glucose levels over normal or hyperglycemia are indicated by impaired glucose tolerance (IGT) or impaired fasting blood glucose levels (IFG). In contrast to IFG, which occurs when blood glucose levels often exceed normal blood glucose concentrations above 7 mmol/L, IGT is characterized by blood glucose levels that are greater than 11 mmol/L 2 hours after a 75-g oral glucose load. In recent investigations of diabetes as a cause of male factor infertility, IGF and IGT were employed as diagnostic criteria. They are independent markers for diagnosis of diabetic mellitus (DM). These studies found that diabetes mellitus affects sperm motility, sperm counts, semen volume, and normal sperm morphology in addition to impairing erectile and ejaculatory function (Alves et al., 2013a; Alves et al., 2013b). There will be more men of reproductive age than ever before as the prevalence of diabetes is expected to reach 422 million people worldwide in 2014, and the number of children and adolescents with the condition is increasing (Wild et al., 2004; Agbaje et al., 2007). Furthermore, it is becoming more widely recognized that male infertility is a sign of declining male health (Eisenberg et al., 2015), especially when sexual dysfunction is prevalent (Lotti et al., 2016).

**FIGURE 1:**
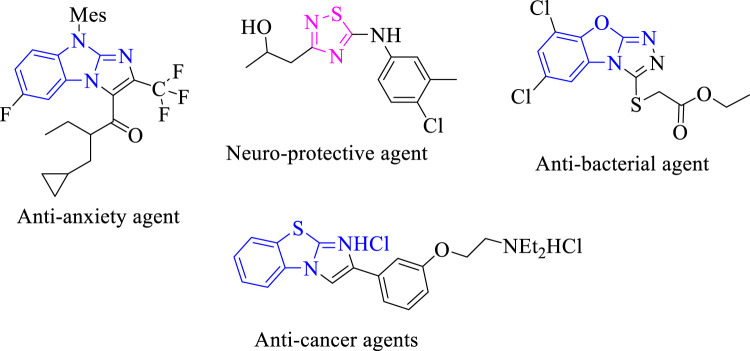
Biological significance of fused heterocyclic compounds.

It was reported that numerous marketed drugs hold heteroarenes as the core center in their structure (Leeson and Springthorpe, 2007). Among heteroarene-bearing scaffolds, fused heteroarene is known as a prominent entity in numerous biologically active analogs for drug discovery and, hence, received much interest from medicinal chemists due to their presence in several biologically potent drugs. Furthermore, fused heterocyclic compounds displayed interesting biological activities such as benzothiazole fused with imidazole biologically reported as a potent antitumor agent for treatment of lung cancer in humans (Amino et al., 2006). Moreover, triazole fused with the benzoxazole moiety was reported to display an antibacterial profile (Shreedhara et al., 2017). Imidazole fused with benzo imidazole was reported to exhibit antianxiety activity for treatment of neuropsychiatric disorders (Han et al., 2005). Thiadiazole scaffolds as the core center of numerous sulfur-containing five-membered rings were found to have a broad spectrum of biological activities, such as antibacterial and neuroprotective profiles (Perlovich et al., 2012; Fang et al., 2017) (Figure 1).

One of the most difficult areas of drug development, particularly for oral-drug delivery systems, continues to be the enhancement of drug solubility and, consequently, its oral bioavailability. The solubility of poorly water-soluble medications can be improved using a variety of techniques that have been published in the literature. The methods are chosen based on a number of factors, including the qualities of the medicine under consideration, the kind of excipients to be chosen, and the kind of dosage form intended. Poor solubility and a sluggish rate of drug dissolution in aqueous gastrointestinal fluids are common causes of insufficient bioavailability for poorly water-soluble drugs. Especially for class II (low solubility and high permeability) drugs, increase in the drug’s solubility and rate of dissolution in gastrointestinal fluids may enhance bioavailability. Increasing solubility also enhances the bioavailability of Biopharmaceutical Classification System (BCS) class II medicines because, rather than absorption, the rate-limiting processes for BCS class II drugs are solubility in the stomach fluid and drug release from the dose form (Savjani et al., 2012).

**FIGURE 2:**
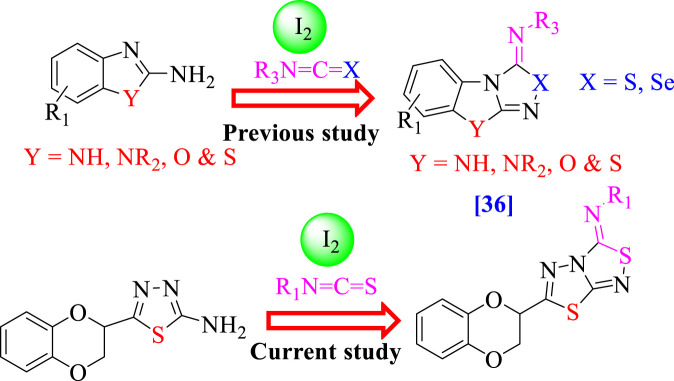
Rationale of the current study.

Consequently, the broad range of biological activities possessed by these fused heterocyclic compounds, especially benzimidazole/benzoxazole/benzothiazole fused with thiadiazole/selenadiazole (Chen et al., 2020) (Figure 2) and further *α*-glucosidase and *α*-amylase inhibition properties possessed by thiadiazole analogs (Taha et al., 2017; Khan et al., 2022a), motivated us to design and synthesize a new class of heterocyclic compounds based on fused heteroarene such as 1,3,4-thiadiazole fused with 1,2,4-thiadiazole incorporating the benzodioxine moiety as an antidiabetic agent for treatment of the diabetic patient. Keeping in mind the biological importance of fused heterocyclic compounds, we deliberately carried out the synthesis of 1,4-benzodioxin-based 1,3,4-thiadiazole-fused-[1,2,4]-thiadiazole analogs and studied their α-amylase and α-glucosidase activities. Studies on the structure–activity connection and molecular docking were excluded to dilute the findings.

## 2 Results and discussion

### 2.1 Chemistry

**SCHEME 1:**
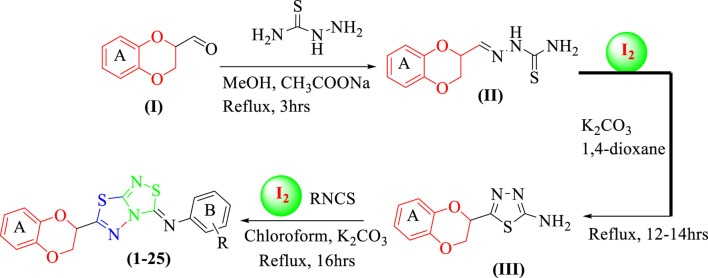
Synthesis of 1,4-benzodioxin-based fused bis-thiadiazole analogs **(1–25)**

Twenty-five analogs of 1,4-benzodioxin-based 1,3,4-thiadiazole-fused-[1,2,4]-thiadiazole were synthesized (Table 1). First of all, 2-formyl 1,4-benzodioxine **(I,** 1 equivalent**)** was reacted with thiosemicarbazide (1 equivalent) in methanol (10 ml) under the catalytic action of sodium acetate (0.8 mmol) to afford 1,4-benzodioxine-based thiosemicarbazone as the substrate **(II)**. This intermediate was cleaned with hexane and deionized water to eliminate all impurities. Substrate **(II,** 1 equivalent**)** was subjected to an iodine-mediated (0.6 mmol) cyclization in 1,4-dioxane (10 ml) in the presence of potassium carbonate (0.8 mmol), and the resulting mixture was stirred for 12–14 h under reflux to obtain 1,4-benzodioxine-based 1,3,4-thiadiazole-2-amine as an intermediate **(III**). In the last step, substrate **(III,** 1 equivalent) further undergoes [3+2] iodine-mediated (0.6 mmol) oxidative cyclization with different phenylisothiocyanates (1 equivalent) in chloroform (10 ml) and potassium carbonate (0.8 mmol), and the resulting mixture was put on reflux for 16 h. It was reacted with a 5% sodium thiosulfate solution after being cooled to 25°C, followed by extraction with DCM (10 ml) to obtain targeted 1,4-benzodioxin-based 1,3,4-thiadiazole-fused-[1,2,4]-thiadiazole analogs **(1–25)** in an appropriate yield. The reaction progress was checked by employing a TLC plate. Different spectroscopic tools, including HREIMS and NMR, were employed to find the exact structure of the afforded analogs (Scheme 1).

**TABLE 1:** Different substituents and inhibitory potentials *(in vitro)* of newly prepared analogs of 1,3,4-thiadiazole-fused-[1,2,4]-thiadiazole incorporating 1,4-benzodioxine skeleton against *α*-amylase and *α*-glucosidase enzymes.

Compound	Ring B	*α*-amylase (μM ± SEM)	*α*-glucosidase (μM ± SEM)
1	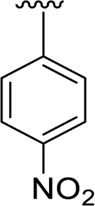	1.60 ± 0.20	9.80 ± 0.20
2	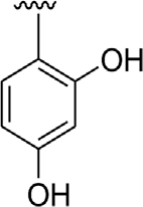	3.40 ± 0.10	4.10 ± 0.10
3	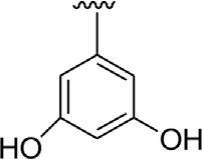	4.40 ± 0.10	5.10 ± 0.10
4	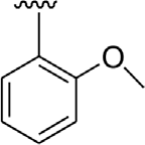	14.20 ± 0.30	15.60 ± 0.30
5	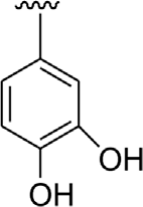	1.10 ± 0.10	1.30 ± 0.10
6	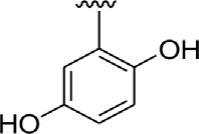	5.20 ± 0.10	6.10 ± 0.10
7	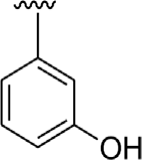	9.20 ± 0.20	10.20 ± 0.20
8	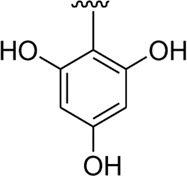	0.90 ± 0.01	1.10 ± 0.01
9	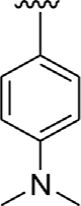	11.10 ± 0.20	11.40 ± 0.20
10	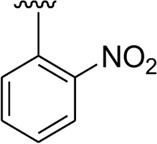	13.60 ± 0.20	14.20 ± 0.20
11	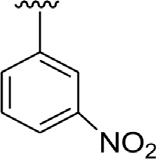	16.20 ± 0.30	17.30 ± 0.30
12	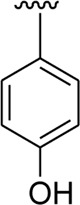	8.20 ± 0.20	8.40 ± 0.20
13	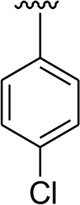	7.50 ± 0.20	5.20 ± 0.10
14	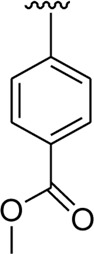	14.30 ± 0.30	15.60 ± 0.30
15	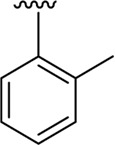	16.10 ± 0.30	17.20 ± 0.30
16	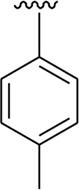	12.20 ± 0.20	13.40 ± 0.50
17	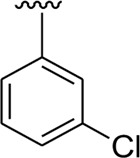	9.60 ± 0.20	8.40 ± 0.20
18	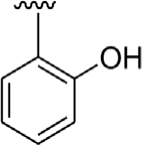	3.20 ± 0.10	2.80 ± 0.10
19	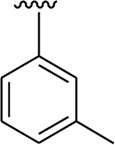	19.20 ± 0.40	18.40 ± 0.40
20	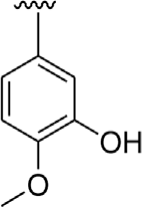	1.60 ± 0.10	1.90 ± 0.10
21	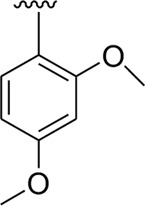	30.80 ± 0.80	29.70 ± 0.40
22	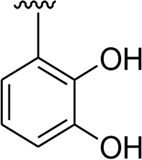	0.70 ± 0.01	0.80 ± 0.01
23	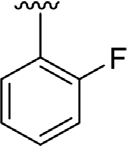	1.80 ± 0.10	1.90 ± 0.10
24	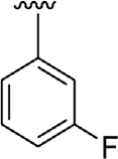	5.30 ± 0.10	5.10 ± 0.10
25	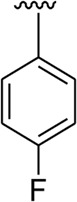	2.10 ± 0.10	2.20 ± 0.10
Standard acarbose		12.80 ± 0.10	12.90 ± 0.10

### 2.2 *In vitro* α-amylase and α-glucosidase inhibitory activities **(1–25)**

**FIGURE 3:**
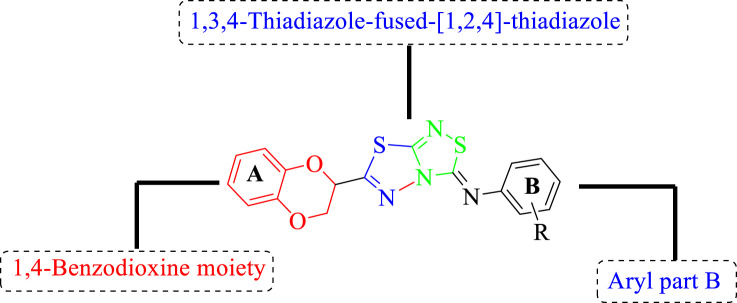
General structure of 1,4-benzodioxin-based 1,3,4-thiadiazole-fused-[1,2,4]-thiadiazole analogs **(1–25)**.

All of the synthetically created hybrid analogs based on 1,3,4-thiadiazole-fused-1[1,2,4]-thiadiazole containing the 1,4-benzodioxine ring were generated and then evaluated for their (*in vitro*) inhibitory profiles for α-amylase and α-glucosidase. The entire analogs illustrated remarkable potency against α-amylase and α-glucosidase having IC_50_ values ranging from 0.70 ± 0.01 to 30.80 ± 0.80 μM (α-amylase**)** and 0.80 ± 0.01 to 29.70 ± 0.40 μM (α-glucosidase) when compared to standard acarbose drugs. It was suggested by SAR studies that each part of the synthesized analogs such as benzodioxine ring A, 1,3,4-thiadiazole-fused-[1,2,4]-thiadiazole and aryl ring B actively participated in the activity, and any variation around ring B resulted to different activity (Figure 3).

#### 2.2.1 Structure–activity relationship for α-amylase activity **(1–25)**

**FIGURE 4:**
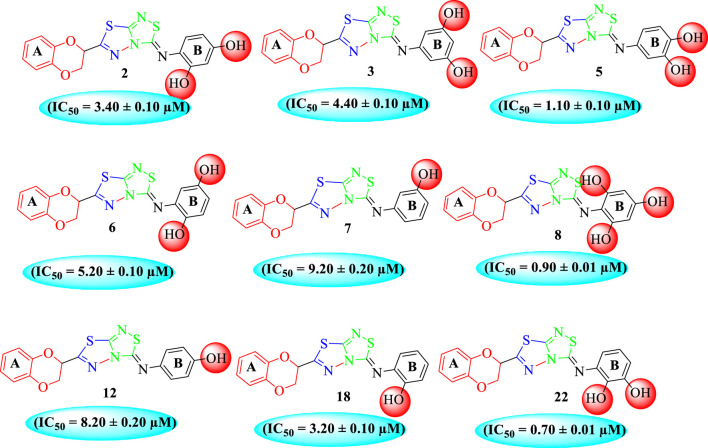
SAR studies of compounds 2, 3, 5, 6, 7, 8, 12, 18, and 22.

Among the synthesized analogs, the analogs bearing tri-hydroxy and di-hydroxy substitutions around ring B have emerged as potent inhibitors of α-amylase among the series. By comparing analog **22** (IC_50_ = 0.70 ± 0.01 μM) bearing di-hydroxy substitutions at the 2,3-position of ring B showed excellent α-amylase activity when compared to other analogs **2** (IC_50_ = 3.40 ± 0.10 μM), **3** (IC_50_ = 4.40 ± 0.10 μM), **5** (IC_50_ = 1.10 ± 0.10 μM), **6** (IC_50_ = 5.20 ± 0.10 μM),**7** (IC_50_ = 9.20 ± 0.20 μM), **12** (IC_50_ = 8.20 ± 0.20 μM), **18** (IC_50_ = 3.20 ± 0.10 μM), and analog **8** (IC_50_ = 0.90 ± 0.01 μM bearing tri-hydroxy substitutions at 2,4,6-position of phenyl ring) under the positive control of standard acarbose (IC_50_ = 12.80 ± 0.10 μM). This enhanced activity of analog **22** showed the presence of attached di-hydroxy groups at the 3,4-position, which could enhance the inhibitory potentials through active participation in conventional hydrogen bonding with enzyme active residues. Moreover, analog **8** bearing tri-hydroxy substitutions at the 2,4,6-position of ring B was found to be second most potent and displayed enhanced activity than di-hydroxy substitutions bearing analogs **2, 3, 5,** and **6,** indicating that any variation in the position of substitution around ring **B** may result to a different activity. In addition, analogs **2, 3,** and **5** bearing di-hydroxy substitution at phenyl ring B displayed superior activity than analogs **7** and **12** bearing only mono-hydroxy substitution. This enhanced activity of analogs **2, 3,** and **5** was due to greater numbers of the attached hydroxy groups that might have more tendencies to interact with the active site of the enzyme. However, by comparing structurally similar analogs **7, 18,** and **12** having a mono-hydroxy substitution at *meta*-, *ortho-*, and *para-*position of the phenyl ring, among these analogs, **18** was more potent than its counterparts **7** and **12**. This difference in activity shows that alteration in the position of substituted around phenyl ring B greatly affects the activity (Figure 4).

**FIGURE 5:**
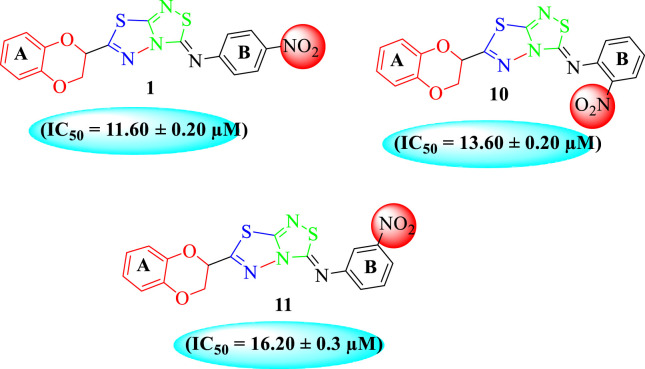
Structure–activity relationship (SAR) for compounds 1, 10, and 11.

In the same way, by comparing *para-*nitro moiety-bearing analog **1** with analogs **10** and **11** that hold the nitro moiety at *ortho*- and *meta*-position of the phenyl ring, compound **1** displayed better activity than its structurally similar compounds **10** and **11**. This indicates that inhibitory potentials were greatly affected by altering the position of the electron-withdrawing nitro group around the phenyl ring attached to the thiadiazole-fused-thiadiazole skeleton (Figure 5).

**FIGURE 6:**
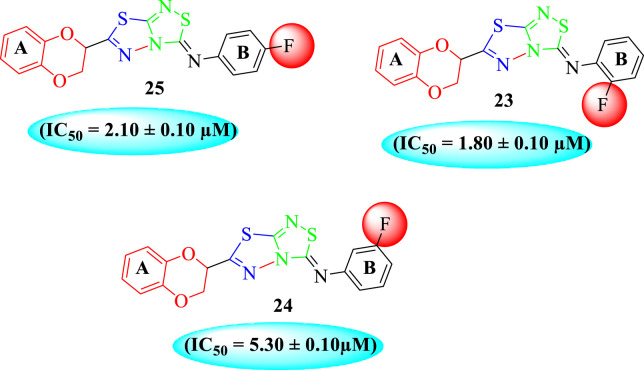
Structure–activity relationship (SAR) of compounds 23, 24, and 25.

The fluoro-substituted analogs show remarkable potency when compared to the –Cl moiety-bearing analogs. This was due to the strong EW nature of the –F moiety than the –Cl group which has a lower tendency of electron-withdrawing effect. Among fluoro-substituted analogs such as **23** (IC_50_ = 1.80 ± 0.10 μM), **24** (IC_50_ = 5.30 ± 0.10 μM), and **25** (IC_50_ = 2.10 ± 0.10 μM), the analogs **23** and **25** bearing the –F moiety at the *ortho*- and *para-*position of the phenyl ring showed superior potency when compared to its counterpart **24** that bears the –F moiety at *meta*-position. The diverse potency of these analogs was due to different attachment of the –F moiety around aryl ring B (Figure 6).

It was shown from SAR studies that attachment of the substituent of either weak electron-withdrawing or weak electron-donating nature such as –OCH_3_ and –CH_3_ groups resulted in decreased inhibitory potentials. Moreover, it was also noted that analogs bearing either substituent of the strong electron-withdrawing nature such as –NO_2_ and –F or substituents capable of forming hydrogen bonding such as –OH groups enhanced the inhibitory potentials. In addition, alteration in position, number/s, and nature of the substituent may result in diverse inhibitory potentials.

#### 2.2.2 Structure–activity relationship for α-glucosidase activity **(1–25)**

**FIGURE 7:**
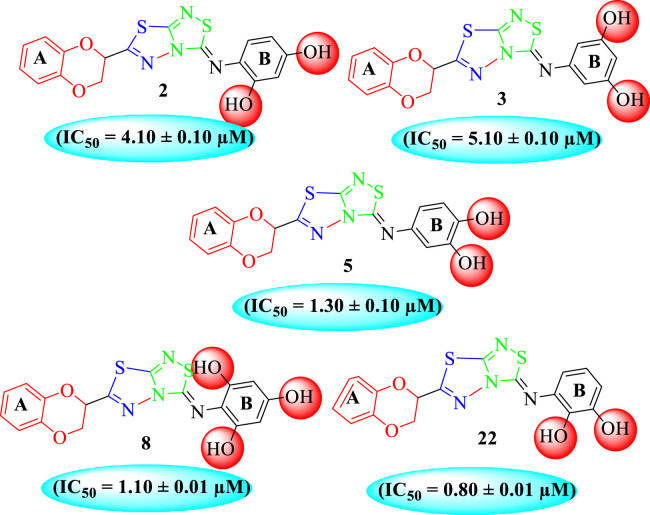
Structure–activity relationship (SAR) of compounds 2, 3, 5, 8, and 22.

It was noteworthy that the attachment of substituents that are capable of interactions in a better way with the active site of α-glucosidase such as –OH groups at appropriate positions enhanced the inhibitory potentials against **α-**glucosidase. Therefore, analog **22** bearing di-hydroxy substitutions at the 2, 3-position of the phenyl ring emerged as most active against α-glucosidase and showed ten-fold more potency than standard acarbose drug. This analog **22** having di-hydroxy substitutions shows better activity than other analogs **2, 3,** and **5** bearing di-hydroxy substitutions at various positions of ring B, indicating that the hydroxyl group in greater numbers enhanced the inhibition profile. Compound **8** having tri-hydroxy moieties at 2,4,6-positions of the phenyl ring shows superior inhibitory activity when compared to other di-hydroxy group-bearing analogs such as **2** (bearing di-hydroxy at 2,4-position), **3** (bearing di-hydroxy at 3,5-position), and **5** (bearing di-hydroxy at 3,4-position). This superior activity of analog **8** was due to the greater number/s of attached hydroxyl moieties at the 2,4,6-position of phenyl ring B (Figure 7).

**FIGURE 8:**
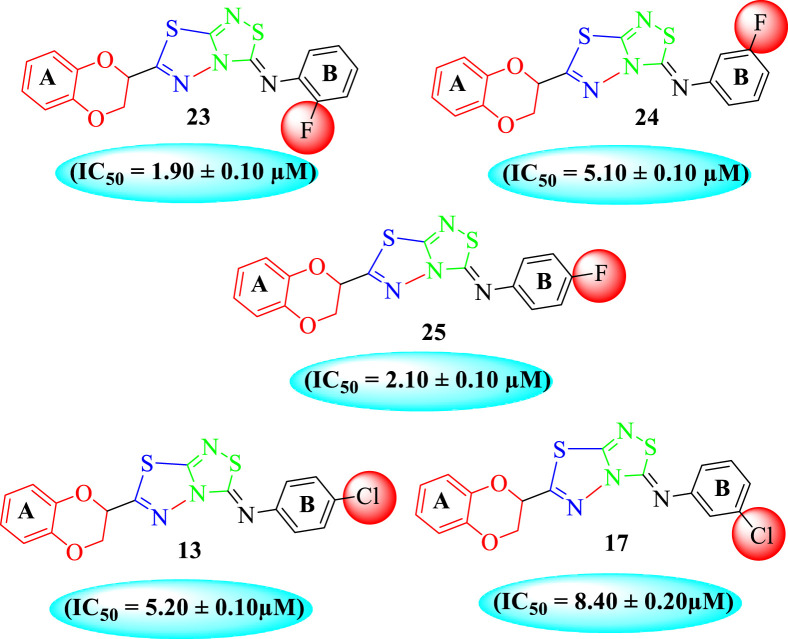
Structure–activity relationship (SAR) of compounds 23, 24, 25, 13, and 18.

Among halogen-substituted analogs, the compounds bearing the –F moiety such as **23, 24,** and **25** at *ortho-, meta-,* and *para*-position of the phenyl ring showed better activity than analogs such as **13** and **17** bearing the –Cl moiety at *para*- and *meta*-position of ring B, indicating that stronger electron-withdrawing effect shows that the moiety has more tendency of interactions with the active site of the enzyme and, therefore, enhanced the activity. Furthermore, comparing compound **23** bearing the –F moiety at the *ortho*-position of ring B showed superior potency in comparison to analogs **24** and **25** which also hold similar substituent like the –F group at the *meta*- and *para*-position of phenyl ring B. This difference in potency of these analogs was due to the different positions of the attached electron withdrawing –F group around ring B (Figure 8).

**FIGURE 9:**
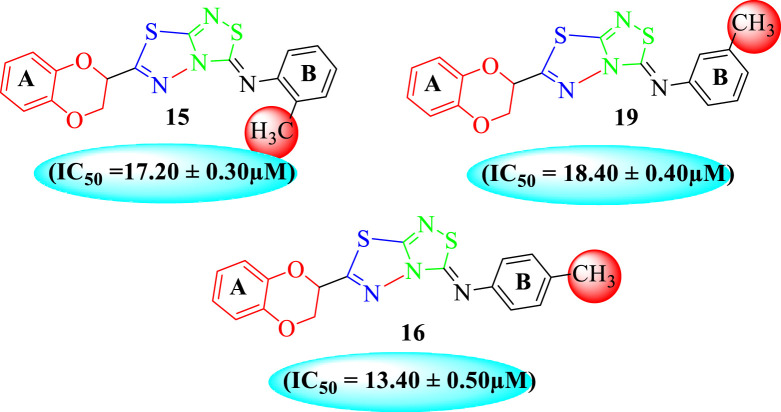
Structure–activity relationship (SAR) of compounds 15, 16, and 19.

Analog **16** (IC_50_ = 13.40 ± 0.50 μM) holding methyl substitution at the *para*-position of ring B displayed superior potency in comparison to analogs **15** (IC_50_ = 17.20 ± 0.30 μM) and **19** (IC_50_ = 18.40 ± 0.40 μM) that also hold methyl groups at the *ortho*- and *meta*-position of phenyl ring B. This difference in potency of these analogs was due to the different positions of the attached electron donating –CH_3_ groups around ring B (Figure 9).

Overall, it was concluded that shifting the positions of substituents around aryl ring B had a significant impact on the inhibitory potentials against the α-glucosidase enzyme. It was also noted that alteration in inhibitory potentials was observed by attachment of substituents of different nature in different number/s around the phenyl ring linked to 1,3,4-thiadiazole-fused-[1,2,4]-thiadiazole incorporating the 1,4-benzodioxine skeleton.

### 2.3 Molecular docking study

**FIGURE 10:**
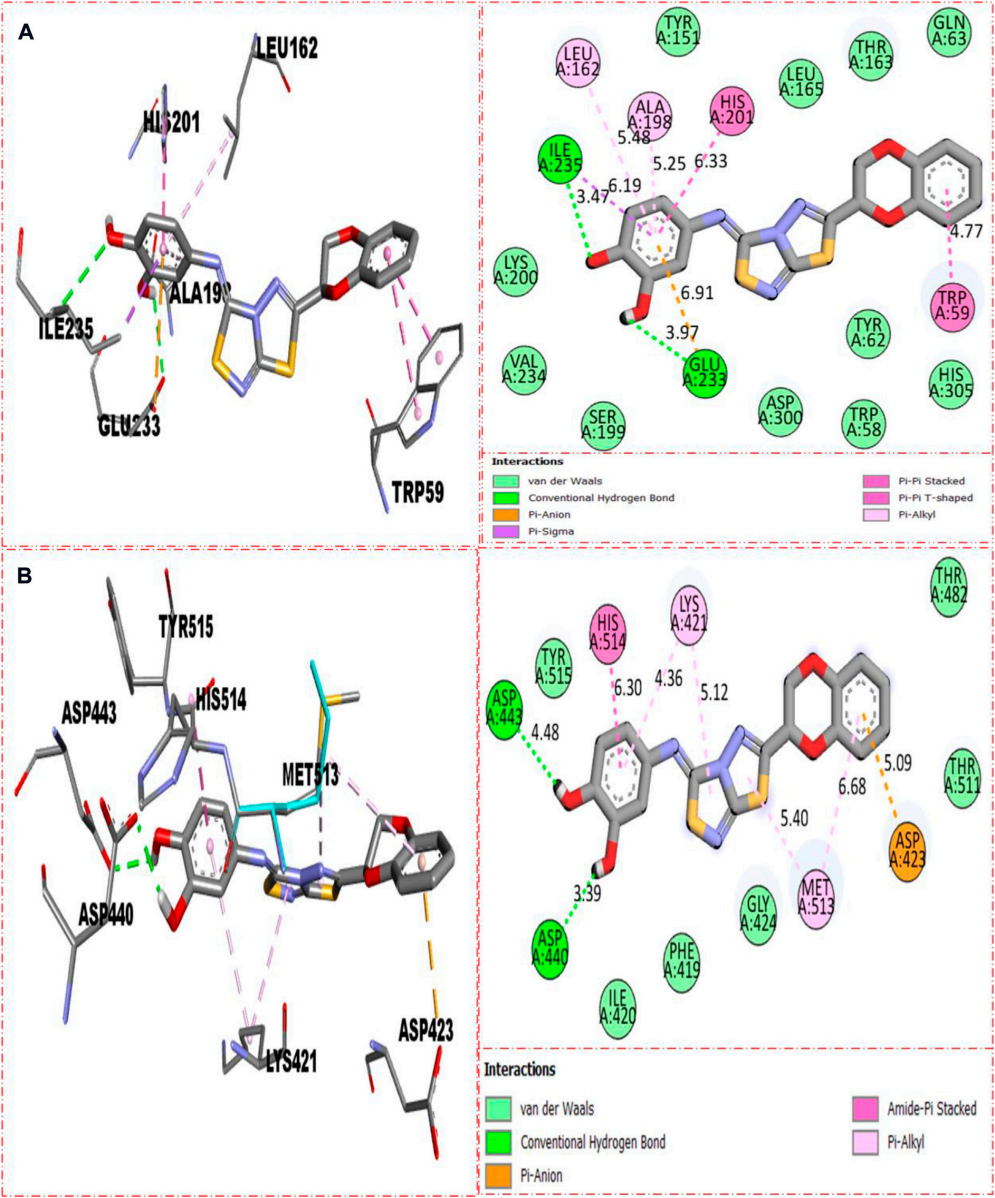
Represent the binding interactions of the most active compound **22**. **(A)** represents compound **22** against α-amylase, while **(B)** denotes the same compound **22** against α-glucosidase enzyme and its 3D and 2D diagram.

The molecular docking had been conducted on α-amylase and α-glucosidase enzymes for most active compounds **22, 8,** and **5** to explore the binding sites of newly prepared analogs and how they interact with the active site of both these targeted enzymes. From the *in vitro* study, it was concluded that compound **22** was found as the most potent inhibitor of both these targeted enzymes due to active participation of both attached hydroxyl groups in strong interactions such as hydrogen bonding. Therefore, keeping in mind the best inhibitory potentials of active analog **22**, it is further subjected to a molecular docking study to explore its binding site, and the result obtained shows that compound **22** established several key interactions, such as Glu233 (H-bonding & pi–anion), Ile235 (pi–sigma and H-bonding), Leu162 and Ala198 (pi–pi T-shaped), His201 and Trp59 (pi–pi stacking), and numerous van der Waals interactions, when evaluated against α-amylase as shown in Figure 10A. While against α-glucosidase, it also furnished numerous key interactions, including Asp440 (CHB), Asp443 (CHB), His514 (amide–pi stacking), Lys421 (pi–alkyl), Met513 (pi–alkyl), and Asp423 (pi–anion), and several other residues interact through van der Waals interactions as shown in Figure 10B.

**FIGURE 11:**
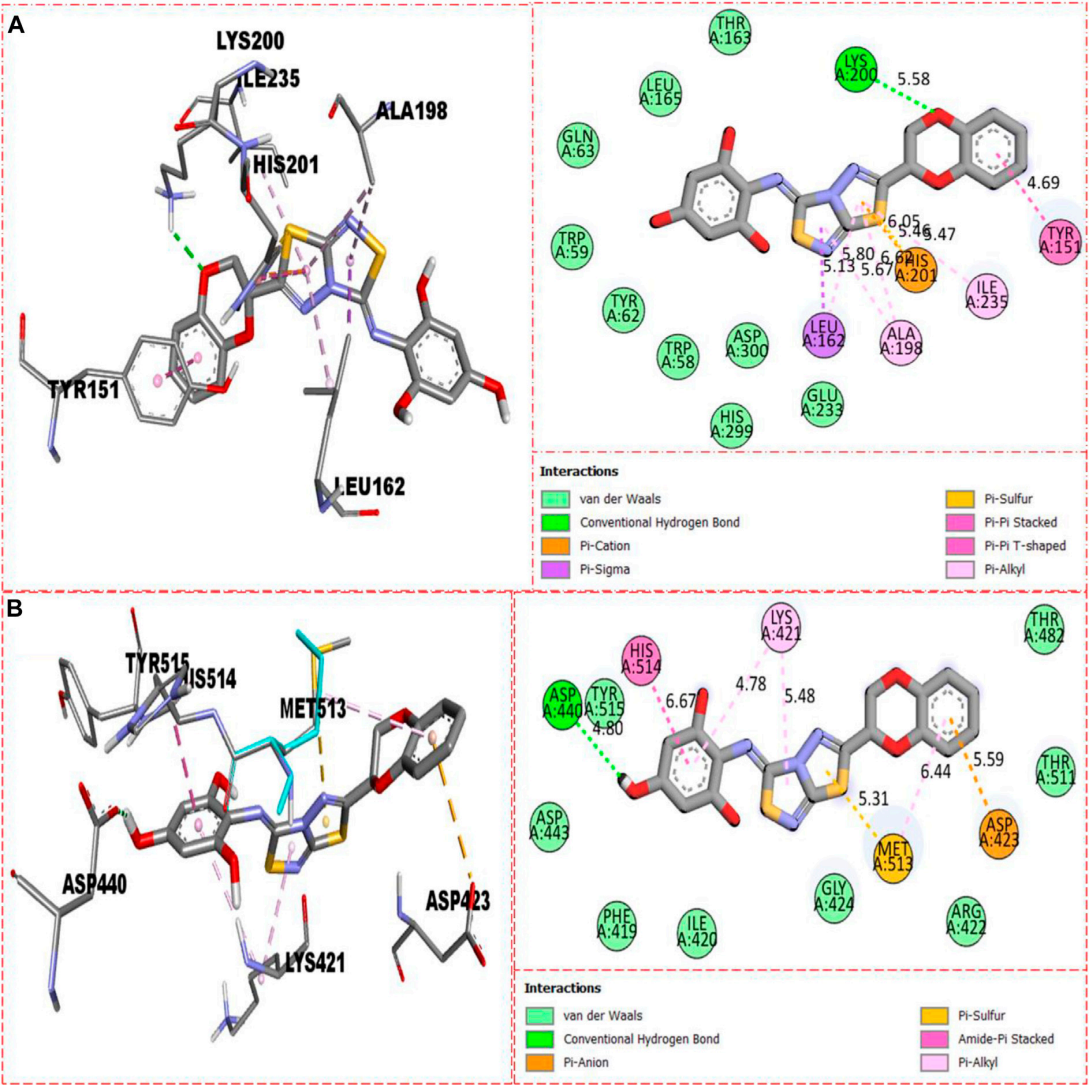
Represents the binding interactions of the second most active compound **8**. **(A)** for compound **8** against α-amylase, while **(B)** for the same compound **5** against α-glucosidase enzyme and its 3D and 2D diagram.

For second-most active compound **8** bearing tri-hydroxyl groups at the 2,4,6-position of the aryl ring which can enhance its inhibitory potentials, this compound **8** when evaluated against α-amylase enzyme showed somewhat fewer interactions than compound **22**, and, hence, its inhibitory potentials were found less than those of compound **22,** which showed better binding interactions. Against α-amylase, this compound **8** revealed several interactions such as Lys200 (CHB), Tyr151 (pi–pi stacking), His201 (pi–sulfur & pi–cation), Ile235 (pi–alkyl), Ala198 (pi–pi T-shaped), Leu162 (pi–sigma & pi–alkyl), and van der Waals interactions (Figure 11A), while against α-glucosidase, it furnished numerous key interactions involving Asp423 (pi–anion), Met513 (pi–sulfur), Asp440 (CHB), His514 (amide–pi stacking), Lys421 (pi–alkyl), and numerous van der Waals interactions as shown in Figure 11B.

**FIGURE 12:**
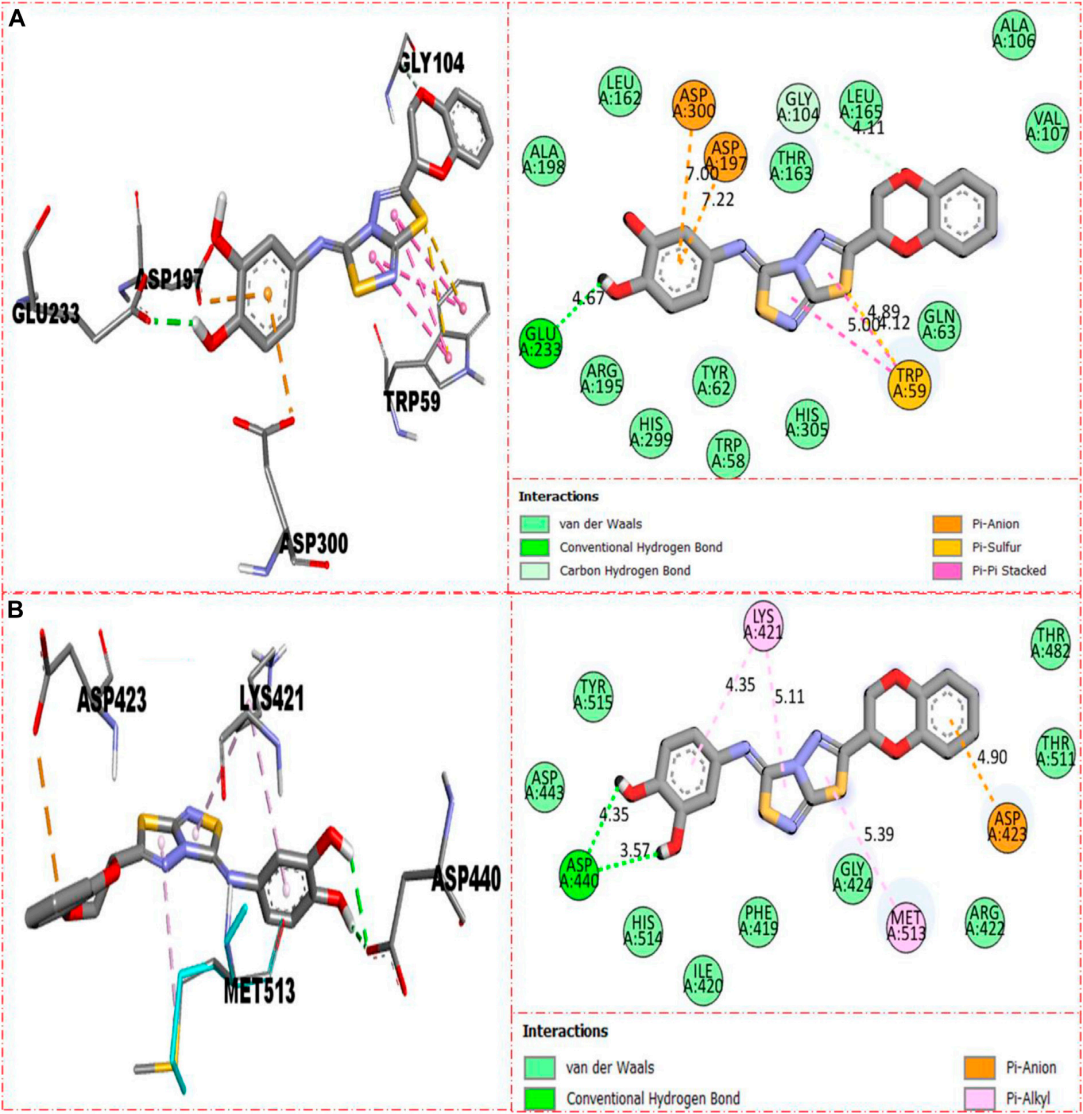
Represent the binding interactions of the third most active compound **5**. **(A)** for compound **5** against α-amylase, while **(B)** for the same compound **5** against α-glucosidase enzyme and its 3D and 2D diagram.

Compound **5** was recognized as the third most active compound bearing di-hydroxy moieties at the 3,4-position of the aryl ring attached to fused thiadiazole rings. However, this compound **5** was found less potent than analog **22,** which is structurally equivalent to it. It might be owing to the different positions of attached di-hydroxy groups around the aryl ring. The result obtained from the docking study shows that compound **5** exhibits various key interactions with the active residue of α-amylase, including Glu233 (CHB), Asp300 and Asp197 (pi–anion), Gly104 (carbon–hydrogen bond), Trp59 (pi–sulfur & pi–pi stacking), and van der Waals interactions (Figure 12A), while against α-glucosidase, it also displayed numerous interactions such as Asp440 (CHB), Asp423 (pi–anion), Met513 and Lys421 (pi–alkyl), and van der Waals interactions (Figure 12B).

## 3 Materials and methods

### 3.1 General information

All biological and chemical components were bought from Merck KGaA and Sigma-Aldrich Chemical Corp., both of St. Louis, Missouri, United States (Merck, Darmstadt, Germany). Using a Silica Gel 60 F254 TLC plate, thin-layer chromatography (TLC) was used to assess the purity of all intermediate and necessary chemicals (Merck KGaA, Darmstadt, Germany). A Stuart Melting Point Apparatus SMP30 was used to measure melting points (Staffordshire, United Kingdom). The ^1^H-NMR and ^13^C-NMR spectra were captured using a 500-MHz NMR instrument in units relative to a deuterated solvent serving as an internal reference. The electron impact high-resolution mass spectra (60 eV) were conducted on a Finnigan MAT-311A instrument (Germany). The chromatogram was shown using UV light from Schimazdu, Germany, with a wavelength of 254/365.

### 3.2 General procedure for the synthesis of 1,4-benzodioxine-based thiadiazole-fused-thiadiazole derivatives **(1–25)**

#### 3.2.1 Synthesis of (E)-2-((2,3-dihydrobenzo[b][1,4]dioxin-2-yl)methylene)hydrazine-1-carbothioamide **(II)**

Thiosemicarbazide (1 equivalent), sodium acetate (0.8 mmol), and benzodioxine **I** (1 equivalent) were stirred together in 10 ml of methanol followed by addition of a formyl group containing benzodioxine **I** to it. A rotary evaporator was used to evaporate the solvent after the reaction mixture was agitated at 25°C for 3 h. The desired benzodioxine-based thiosemicarbazone **II** was produced in excellent yield after the residue was triturated with ethyl acetate/diethyl ether (5:95).

#### 3.2.2 Synthesis of 5-(2,3-dihydrobenzo[b][1,4]dioxin-2-yl)-1,3,4-thiadiazol-2-amine **(III)**

Further dissolving the intermediate **II** (1 equivalent) in 1,4-dioxane (10 ml), potassium carbonate (0.8 mmol) and iodine (0.6 mmol) were successively added. In a nitrogen environment, the reaction mixture was heated to a reflux temperature until the formation of a new entity was complete (TLC was used to monitor the progress, 12–14 h). It was then brought to room temperature, treated with 20 ml of 5% Na_2_S_2_O_3_, and extracted using EtOAc (3 × 15 ml). Over anhydrous sodium sulfate, the mixed organic layer was dried and concentrated. In order to produce 2-amino-1,3,4-thiadiazole substrate III in a high yield, the supplied residue was purified using silica gel column chromatography with petroleum ether and EtOAc combination as the eluent.

#### 3.2.3 Synthesis of targeted benzodioxine-based thiadiazole-fused-thiadiazole analogs **(1–25)**

Finally, 2-amino-1,3,4-thiadiazole-based substrate (III, 1 equivalent) further undergoes [3+2] oxidative cyclization on stirring overnight with different substituted phenyl isothiocyanates (1 equivalent) in the presence of molecular iodine (0.6 mmol) and potassium carbonates (0.8 mmol) in chloroform (10 ml) as a solvent to access the targeted 1,3,4-thiadiazole-fused-[1,2,4]-thiadiazole-based benzodioxine analogs **(1–25)** in an appropriate yield. The structures of all the newly discovered analogs were confirmed using spectroscopic techniques, including NMR and HREI-MS.

### 3.3 Assay protocol for the docking study

Utilizing Discovery Studio Visualizer’s (DSV) MGL tool 1.5.7 and AutoDock Vina, a molecular docking study was carried out (Li et al., 2015; Mrabti, 2018; Rao et al., 2021). In this study, the synthetic chemicals were tested against the enzymes glucosidase and amylase. These enzymes’ structures were found in the Protein Data Bank (PDB) by using the search criteria 1b2y & 3w37. The target protein and the prepared ligand were both saved in PDB format after the first step of protein preparation, which involved utilizing DSV to remove water molecules and existing ligands. The process was then continued in AutoDock, where the protein was charged with polar hydrogen, Kollman, and Gasteiger charges. Additionally, the chosen ligand was constructed using a torsion tree to find the root. Also, a configuration file was created and saved in the same docking folder along with the X, Y, and Z axes for both the ligand and protein in PDBQT format. The ligand was then generated in various postures using a command line, yielding a total of nine distinct poses in the PDBQT format. The dock protein and ligand were then opened in DSV to determine the ligand–enzyme active site interaction. The given study summarizes the additional information (Khan et al., 2022b).

## 4 Conclusion

In conclusion, we have designed and then synthesized twenty-five analogs **(1–25)** of thiadiazole-fused-[1,2,4]-thiadiazole-incorporating benzodioxine moiety. The literature-recognized procedure was used to evaluate the synthesized analogs’ ability to inhibit α-amylase and α-glucosidase. When compared to standard acarbose (IC_50_ = 12.80 ± 0.10 μM), (IC_50_ = 12.90 ± 0.10 μM), all analogs demonstrated a diverse range of efficacy with IC_50_ values ranging from 0.70 ± 0.01 to 30.80 ± 0.80 μM (against α-amylase) and 0.80 ± 0.01 to 29.70 ± 0.40 μM (α-glucosidase). The structure–activity relationship was carried out for all analogs based on substitution pattern around the aryl ring, and it was concluded from SAR studies that analogs holding hydroxyl moieties around the aryl ring enhanced the enzymatic activities against both these targeted enzymes *via* the formation of a strong conventional hydrogen bond with enzyme active sites. Additionally, a molecular docking study was performed on the potent analogs to determine the best way for them to interact with the active residues of the intended enzymes. The results revealed that these compounds provide a number of important interactions with the enzyme active site, increasing the enzymes’ enzymatic activities. Additionally, a number of spectroscopic techniques, including HREI-MS, ^13^C-NMR, and ^1^H-NMR, were employed to investigate the precise structure of the synthesized analogs.

## Data Availability

The original contributions presented in the study are included in the article/Supplementary Material; further inquiries can be directed to the corresponding authors.
