# Supplementary material for: Molecular iodine-promoted oxidative cyclization for the synthesis of 1,3,4-thiadiazole-fused- [1,2,4]-thiadiazole incorporating 1,4-benzodioxine moiety as potent inhibitors of α-amylase and α-glucosidase: In vitro and in silico study
Source: Front Chem. 2022 Oct 6;10:1023316. doi: 10.3389/fchem.2022.1023316 (PMC9627624; doi:10.3389/fchem.2022.1023316)
Supplement: Supplementary file 1 [file DataSheet1.PDF]

Molecular iodine-promoted oxidative cyclization for the synthesis of 1,3,4-thiadiazole-fused-[1,2,4]-thiadiazole incorporating 1,4-benzodioxine moiety as potent inhibitors of α-amylase and α-glucosidase: *In vitro* and *in silico* study

Rafaqat Hussain[1], Mazloom Shah[2], Shahid Iqbal[3]*, Wajid Rehman[1]*, Shoaib Khan[1], Liaqat Rasheed[1], Haseena Naz[1], Hanan A. Al-ghulikah[4], Eslam B. Elkaeed[5], Rami Adel Pashameah[6], Eman Alzahrani[7], Abd-ElAziem Farouk[8]

[1]*Department of Chemistry, Hazara University, Mansehra-21300, Khyber Pakhtunkhwa, Pakistan*

[2]*Department of Chemistry, Abbottabad University of Science and Technology (AUST) Abbottabad, Pakistan.*

[3]*Department of Chemistry, School of Natural Sciences (SNS), National University of Science and Technology (NUST), H-12, Islamabad, 46000, Pakistan.*

[4]*Department of Chemistry, College of Sciences, Princess Nourahbint Abdulrahman University, P.O. Box 84428, Riyadh 11671, Saudi Arabia.*

[5]*Department of Pharmaceutical Sciences, College of Pharmacy, AlMaarefa University, Riyadh 13713, Saudi Arabia.*

[6]*Department of Chemistry, Faculty of Applied Science, Umm Al-Qura University, Makkah 24230, Saudi Arabia.*

[7]*Department of Chemistry, College of Science, Taif University, P.O. Box 11099, Taif 21944, Saudi Arabia.*

[8]*Department of Biotechnology College of Science, Taif University, P.O. Box 11099, Taif 21944, Saudi Arabia.*

To whom corresponding should be addressed

shahidgcs10@yahoo.com(Shahid Iqbal) and sono_waj@yahoo.com. (Wajid Rehman)

3.4. α-Glucosidase inhibition assay

The α-glucosidase inhibition activity was performed with slight modifications [43]. A total volume of 100 μL reaction mixture contained, 70 μL 50 mM phosphate buffer pH 6.8, 10 μL (0.5 mM in methanol) test compound, followed by the addition of 10 μL (0.057 units, Sigma Inc.) enzyme solution in the buffer. The contents were mixed, pre-incubated for 10 min at 37 ◦C and pre-read at 400 nm. The reaction was initiated by the addition of 10 μL of 0.5 mM substrate (p-nitrophenyl glucopyranoside, Sigma Inc.). After 30 min of incubation at 37 ◦C, the absorbance of p-nitrophenol was measured at 400 nm using the Synergy HT 96-well plate reader, BioTek, USA. Acarbose was used as positive control. All experiments were carried out in triplicates (mean ± SEM, n = 3). Percent inhibition was calculated by the following equation: Inhibition (%) = (Abs of Control-Abs of Test/Abs of Control) × 100 Active compound solutions were

suitably diluted and their inhibition studies were determined. Data obtained was used for the determination of IC50 values (concentration at which there is 50 % enzyme inhibition) using EZ-Fit Enzyme Kinetics Software (Perrella Scientific Inc. Amherst, USA).

3.5. α-Amylase inhibition assay

The **α**-amylase inhibitory activity was determined by an assay modified from Kwon, Apostolidis and Shetty. A volume of 500 mL of a-amylase solution (0.5 mg/mL) in 0.2 mM phosphate buffer (pH 6.9) and 500 mL of test sample (100, 200, 400, 800, 1000 mg/mL) were incubated for 10 min at 25^0C. After pre-incubation, 1% starch solution (500 mL) in 0.02 M sodium phosphate buffer (pH 6.9) was added to each tube and incubated at 25^0C for 10 min. 1mL of dinitrosalicylic acid color reagent was then added to the reaction mixture and the tubes were incubated in boiling water for 5 min, and finally cooled to room temperature. The solutions were diluted by adding 10mL distilled water and the absorbance was measured at 540 nm [44]. The percentage inhibition was calculated as illustrated in given formula.

$$\%\text{inhibition} = (\text{Absorbance}_{\text{control}} — \text{Absorbance}_{\text{sample}})/\text{Absorbance}_{\text{control}} \times 100$$

3.6. Spectral analysis

3.6.1. (E)-2-((2,3-dihydrobenzo[b][1,4]dioxin-2-yl)methylene)hydrazine-1-carbothioamide (II)

Yield: 95%; mp 133-134°C; 1H-NMR (500MHz, DMSO-d_6): δ 12.36 (s, 1H, NH), 10.23 (s, 2H, -NH_2), 9.88 (s, 1H, HC=N), 5.23 (t, J = 5.6 Hz, 1H, benzodioxine-H-2), 5.34 (d, J = 5.7 Hz, 2H, benzodioxine-H-3), 7.23 (dd, J = 6.8, 1.9Hz, 2H, benzodioxine-H-5/H-8), 6.98-7.08 (m, 2H, benzodioxine-H-6/H-7). ^{13}C-NMR (125 MHz, DMSO-d_6): δ 185.5, 155.5, 144.4, 140.2, 123.4, 121.2, 118.3, 115.5, 85.7, 70.3; HREI-MS: *m/z* calcld for $C_{10}H_{11}N_3O_2S$ $[M]^+$ 237.0535, Found 237.0527.

3.6.2. 5-(2,3-dihydrobenzo[b][1,4]dioxin-2-yl)-1,3,4-thiadiazol-2-amine (III)

Yield: 81%; mp 151-152°C; ^{1}H-NMR (500MHz, DMSO-d_6): δ 11.13 (s, 2H, -NH_2), 5.83 (t, J = 7.6 Hz, 1H, benzodioxine-H-2), 5.54 (d, J = 7.5 Hz, 2H, benzodioxine-H-3), 7.34 (dd, J = 7.9, 2.4Hz, 2H, benzodioxine-H-5/H-8), 7.22-7.14 (m, 2H, benzodioxine-H-6/H-7). ^{13}C-NMR (125

MHz, DMSO-d_6): δ 174.5, 167.5, 148.7, 143.8, 124.8, 122.8, 119.6, 116.3, 86.4, 71.5; HREI-MS: *m/z* calcld for $C_{10}H_9N_3O_2S$ $[M]^+$ 235.0388, Found 235.0373.

3.6.3. (Z)-6-(2,3-dihydrobenzo[b][1,4]dioxin-2-yl)-N-(4-nitrophenyl)-3H-[1,3,4]thiadiazolo[2,3-c][1,2,4]thiadiazol-3-imine (1)

Yield: 68%; mp 210-211°C; ^{1}H-NMR (500 MHz, DMSO-d_6): δ 8.21 (d, J = 7.3 Hz, 2H, ArH), 8.03 (d, J = 7.1 Hz, 2H, ArH), 7.31 (d, J = 6.7 Hz, 2H, ArH), 7.20 (d, J = 6.5 Hz, 2H, ArH), 5.50 (t, J = 5.7 Hz, 1H, ArH), 5.16 (d, J = 5.5 Hz, 2H, ArH). ^{13}C-NMR (125 MHz, DMSO-d_6): δ 176.5, 166.1, 158.5, 150.2, 144.1, 143.3, 142.7, 139.8, 124.2, 124.2, 124.0, 124.0, 120.9, 120.9, 120.2, 115.0, 93.8; HREI-MS: m/z calcld for $C_{17}H_{11}N_5O_2S_2$ $[M]^+$ 413.0247 Found 413.0242.

3.6.4. (Z)-4-((6-(2,3-dihydrobenzo[b][1,4]dioxin-2-yl)-3H-[1,3,4]thiadiazolo[2,3-c][1,2,4]thiadiazol-3-ylidene)amino)benzene-1,3-diol **(2)**

Yield: 66%; mp 201-202°C; ^{1}H-NMR (500 MHz, DMSO-d_6): δ 10.93 (s, 1H, OH), 10.57 (s, 1H, OH), 7.53 (d, J = 6.8Hz, 1H, ArH), 7.47 (s, 1H, ArH), 7.25 (d, J = 6.4 Hz, 2H, ArH), 7.13 (d, J = 6.5 Hz, 2H, ArH), 6.31 (d, J = 6.7 Hz, 1H, ArH),5.53 (t, J = 5.4 Hz, 1H, ArH), 5.17 (d, J = 5.3 Hz, 2H, ArH). ^{13}C-NMR (125 MHz, DMSO-d_6): δ 177.5, 162.5, 162.2, 158.6, 146.0, 143.3, 142.7, 133.8, 120.9, 120.9, 120.2, 115.0, 111.1, 108.6, 103.7, 93.8, 66.1; HREI-MS: m/z calcld for $C_{17}H_{12}N_4O_4S_2$ $[M]^+$ 400.0293 Found 400.0287.

3.6.5. (Z)-5-((6-(2,3-dihydrobenzo[b][1,4]dioxin-2-yl)-3H-[1,3,4]thiadiazolo[2,3-c][1,2,4]thiadiazol-3-ylidene)amino)benzene-1,3-diol **(3)**

Yield: 70%; mp 200-201°C; ^{1}H-NMR (500 MHz, DMSO-d_6): δ 9.19 (s, 2H, OH), 7.24 (d, J = 6.9 Hz, 2H, ArH), 7.25 (d, J = 6.7 Hz, 2H, ArH), 6.88(d, J = 7.02 Hz, 2H, ArH), 6.37 (s, 1H, ArH), 5.58 (t, J = 5.6 Hz, 1H, ArH), 5.18 (d, J = 5.7 Hz, 2H, ArH). ^{13}C-NMR (125 MHz, DMSO-d_6): δ 176.5, 163.0, 160.0, 158.4, 146.8, 143.3, 142.7, 136.5, 120.9, 120.9, 120.2, 115.0, 107.5, 107.5, 105.9, 93.8, 66.1; HREI-MS: m/z calcld for $C_{17}H_{12}N_4O_4S_2$ $[M]^+$ 400.0293 Found 400.0287.

3.6.6. (Z)-6-(2,3-dihydrobenzo[b][1,4]dioxin-2-yl)-N-(2-methoxyphenyl)-3H-[1,3,4]thiadiazolo[2,3-c][1,2,4]thiadiazol-3-imine **(4)**

Yield: 71%; mp 197-198°C; ^{1}H-NMR (500 MHz, DMSO-d_6): δ 7.79(d, J = 7.01 Hz, 1H, ArH), 7.44 (t, J = 6.9 Hz, 1H, ArH), 7.20 (d, J = 6.7 Hz, 1H, ArH), 7.17 (d, J = 6.6 Hz, 1H, ArH), 7.11 (d, J = 6.4 Hz, 2H, ArH), 7.08 (d, J = 6.5 Hz, 2H, ArH), 5.26 (t, J = 5.6 Hz, 1H, ArH), 5.03 (d, J = 5.4 Hz, 2H, ArH), 3.81 (s, 3H, OCH_3). ^{13}C-NMR (125 MHz, DMSO-d_6): δ 174.5, 157.6, 158.7, 146.0, 143.3, 142.7, 132.0, 131.7, 121.1, 120.9, 120.9, 120.2, 116.9, 115.0, 111.2, 93.8, 66.1, 55.8; HREI-MS: m/z calcld for $C_{18}H_{14}N_4O_3S_2$ $[M]^+$ 398.0502 Found 398.0497.

3.6.7. (Z)-4-((6-(2,3-dihydrobenzo[b][1,4]dioxin-2-yl)-3H-[1,3,4]thiadiazolo[2,3-c][1,2,4]thiadiazol-3-ylidene)amino)benzene-1,2-diol **(5)**

Yield: 69%; mp 201-202°C; ^{1}H-NMR (500 MHz, DMSO-d_6): δ 9.46(s, 2H, OH), 7.28 (s, 1H, ArH), 7.27 (d, J = 7.4 Hz, 1H, ArH), 7.16 (d, J = 6.7 Hz, 2H, ArH), 7.10(d, J = 6.8 Hz, 2H, ArH), 6.76 (d, J = 7.4 Hz, 1H, ArH),5.37 (t, J = 5.6 Hz, 1H, ArH), 5.05 (d, J = 5.6 Hz, 2H, ArH). ^{13}C-NMR (125 MHz, DMSO-d_6): δ 177.5, 158.8, 149.6, 146.8, 146.1, 143.3, 142.7, 131.3, 123.2, 120.9, 120.9, 120.2, 117.4, 116.3, 115.0, 93.8, 66.1; HREI-MS: m/z calcld for $C_{17}H_{12}N_4O_4S_2$ $[M]^+$ 400.0293 Found 400.0287.

3.6.8. (Z)-2-((6-(2,3-dihydrobenzo[b][1,4]dioxin-2-yl)-3H-[1,3,4]thiadiazolo[2,3-c][1,2,4]thiadiazol-3-ylidene)amino)benzene-1,4-diol **(6)**

Yield: 67%; mp 199-200°C; ^{1}H-NMR (500 MHz, DMSO-d_6): δ 10.65 (s, 1H, OH), 9.47 (s, 1H, OH), 7.25 (d, J = 6.3 Hz, 2H, ArH), 7.19 (d, J = 6.1 Hz, 2H,ArH), 7.09 (s, 1H, ArH), 6.74 (d, J = 6.9 Hz, 1H, ArH), 6.67 (d, J = 6.9 Hz, 1H, ArH), 5.38 (t, J = 5.5 Hz, 1H, ArH), 5.13 (d, J = 5.3 Hz, 2H, ArH). ^{13}C-NMR (125 MHz, DMSO-d_6): δ 174.5, 158.5, 153.7, 151.2, 146.0, 143.3, 142.7, 122.9, 120.9, 120.4, 122.2, 119.9, 118.6, 116.3, 115.0, 94.8, 66.1; HREI-MS: m/z calcld for $C_{17}H_{12}N_4O_4S_2$ $[M]^+$ 400.0293 Found 400.0287.

3.6.9. (Z)-3-((6-(2,3-dihydrobenzo[b][1,4]dioxin-2-yl)-3H-[1,3,4]thiadiazolo[2,3-c][1,2,4]thiadiazol-3-ylidene)amino)phenol **(7)**

Yield: 69%; mp 191-192°C; ^{1}H-NMR (500 MHz, DMSO-d_6): δ 9.76 (s, 1H, OH), 7.38 (d, J = 7.2 Hz, 1H, ArH), 7.30 (s, 1H, ArH), 7.23 (d, J = 6.4 Hz, 2H, ArH), 7.14 (t, J = 6.9 Hz, 1H, ArH), 7.13 (d, J = 6.3 Hz, 2H, ArH), 6.91 (d, J = 6.7 Hz, 1H, ArH), 5.40 (t, J = 5.4 Hz, 1H, ArH), 5.12 (d, J = 5.2 Hz, 2H, ArH). ^{13}C-NMR (125 MHz, DMSO-d_6): δ 176.5, 158.6, 158.2, 146.8, 143.3,

142.7, 138.7, 130.2, 121.8, 120.9, 120.9, 120.2, 118.4, 115.0, 114.9, 96.8, 62.1; HREI-MS: m/z calcld for $C_{17}H_{12}N_4O_3S_2$ $[M]^+$ 384.0378 Found 384.0373.

3.6.10. (Z)-2-((6-(2,3-dihydrobenzo[b][1,4]dioxin-2-yl)-3H-[1,3,4]thiadiazolo[2,3-c][1,2,4]thiadiazol-3-ylidene)amino)benzene-1,3,5-triol **(8)**

Yield: 72%; mp 214-215°C; ^{1}H-NMR (500 MHz, DMSO-d_6): δ 10.49 (s, 1H, OH), 10.20 (s, 2H, OH), 7.25 (d, J = 6.7 Hz, 2H, ArH), 7.22 (d, J = 6.8 Hz, 2H, ArH), 7.06 (s, 2H, ArH), 5.50 (t, J = 5.6 Hz, 1H, ArH), 5.12 (d, J = 5.7 Hz, 2H, ArH). ^{13}C-NMR (125 MHz, DMSO-d_6): δ 175.9, 163.9, 163.6, 162.9, 158.6, 143.3, 142.7, 142.3, 120.9, 120.9, 120.2, 115.0, 106.2, 96.3, 96.3, 92.8, 66.1; HREI-MS: m/z calcld for $C_{17}H_{12}N_4O_5S_2$ $[M]^+$ 416.0243 Found 416.0238.

3.6.11. (Z)-4-((6-(2,3-dihydrobenzo[b][1,4]dioxin-2-yl)-3H-[1,3,4]thiadiazolo[2,3-c][1,2,4]thiadiazol-3-ylidene)amino)-N,N-dimethylaniline **(9)**

Yield: 65%; mp 206-207°C; ^{1}H-NMR (500 MHz, DMSO-d_6): δ 7.51 (d, J = 6.9 Hz, 2H, ArH), 7.26 (d, J = 6.3 Hz, 2H, ArH), 7.14 (d, J = 6.3 Hz, 2H, ArH), 6.84 (d, J = 6.8 Hz, 2H, ArH), 5.55 (t, J = 5.7 Hz, 1H, ArH), 5.12 (d, J = 5.3 Hz, 2H, ArH), 3.03 (s, 6H, CH3). ^{13}C-NMR (125 MHz, DMSO-d_6): δ 172.5, 158.9, 151.4, 144.1, 143.3, 142.7, 128.3, 128.3, 123.3, 120.9, 120.9, 120.2, 115.0, 111.9, 111.9, 93.8, 66.1, 42.3, 41; HREI-MS: m/z calcld for $C_{19}H_{17}N_5O_5S_2$ $[M]^+$ 411.0819 Found 411.0813.

3.6.12. (Z)-6-(2,3-dihydrobenzo[b][1,4]dioxin-2-yl)-N-(2-nitrophenyl)-3H-[1,3,4]thiadiazolo[2,3-c][1,2,4]thiadiazol-3-imine **(10)**

Yield: 66%; mp 209-210°C; ^{1}H-NMR (500 MHz, DMSO-d_6): δ 8.14 (d, J = 7.3 Hz, 1H, ArH), 7.97 (d, J = 7.2 Hz, 1H, ArH), 7.66 t, J = 7.2 Hz, 1H, ArH), 7.55 t, J = 7.1 Hz, 1H, ArH), 7.26 (d, J = 6.8 Hz, 2H, ArH), 7.18 (d, J = 6.4 Hz, 2H, ArH), 5.53 (t, J = 5.5 Hz, 1H, ArH), 5.07 (d, J = 5.2 Hz, 2H, ArH),). ^{13}C-NMR (125 MHz, DMSO-d_6): δ 174.5, 158.1, 147.8, 143.3, 143.3, 142.7, 134.9, 131.9, 130.1, 128.4, 124.0, 120.9, 120.9, 120.2, 115.0, 93.8, 66.1; HREI-MS: m/z calcld for $C_{17}H_{11}N_5O_2S_2$ $[M]^+$ 413.0247 Found 413.0242.

3.6.13. ((Z)-6-(2,3-dihydrobenzo[b][1,4]dioxin-2-yl)-N-(3-nitrophenyl)-3H-[1,3,4]thiadiazolo[2,3-c][1,2,4]thiadiazol-3-imine **(11)**

Yield: 69%; mp 208-209°C; ^{1}H-NMR (500 MHz, DMSO-d_6): δ 8.46 (s, 1H, ArH), 8.09 (d, J = 7.4 Hz,1H, ArH), 8.01 (d, J = 7.1, Hz,1H, ArH), 7.73 (t, J = 7.1 Hz,1H, ArH),7.29 (d, J = 6.3 Hz, 2H, ArH), 7.18 (d, J = 6.5 Hz, 2H, ArH), 5.51 (t, J = 5.4 Hz, 1H, ArH), 5.08 (d, J = 5.1 Hz, 2H, ArH). ^{13}C-NMR (125 MHz, DMSO-d_6): δ 174.5, 158.2, 148.0, 143.3, 142.8, 142.7, 134.6, 130.5, 129.7, 126.2, 121.9, 121.6, 120.9, 120.2, 115.0, 93.8, 66.1; HREI-MS: m/z calcld for $C_{17}H_{11}N_5O_2S_2$ $[M]^+$ 413.0247 Found 413.0242.

3.6.14. (Z)-4-((6-(2,3-dihydrobenzo[b][1,4]dioxin-2-yl)-3H-[1,3,4]thiadiazolo[2,3-c][1,2,4]thiadiazol-3-ylidene)amino)phenol **(12)**

Yield: 70%; mp189-190°C; ^{1}H-NMR (500 MHz, DMSO-d_6): δ 9.75 (s, 1H, OH), 7.62 (d, J = 6.8 Hz, 2H, ArH), 7.25 (d, J = 6.2 Hz, 2H, ArH), 7.12 (d, J = 6.9 Hz, 2H, ArH), 6.82 (d, J = 6.7 Hz, 2H, ArH), 5.44 (t, J = 5.6 Hz, 1H, ArH), 5.11 (d, J = 5.2 Hz, 2H, ArH). ^{13}C-NMR (125 MHz, DMSO-d_6): δ 175.0, 162.8, 158.8, 144.1, 143.9, 143.7, 132.6, 131.6, 126.3, 120.9, 120.9, 120.5, 116.0, 116.0, 115.0, 93.8, 66.1; HREI-MS: m/z calcld for $C_{17}H_{12}N_4O_3S_2$ $[M]^+$ 384.0378 Found 384.0373.

3.6.15. ((Z)-N-(4-chlorophenyl)-6-(2,3-dihydrobenzo[b][1,4]dioxin-2-yl)-3H-[1,3,4]thiadiazolo[2,3-c][1,2,4]thiadiazol-3-imine **(13)**

Yield: 74%; mp 204-205°C; ^{1}H-NMR (500 MHz, DMSO-d_6): δ 7.77 (d, J = 7.5 Hz, 2H, ArH), 7.54 (d, J = 7.5 Hz, 2H, ArH), 7.21 (d, J = 6.6 Hz, 2H, ArH), 7.12 (d, J = 6.5 Hz, 2H, ArH), 5.43 (t, J = 5.7 Hz, 1H, ArH), 5.04 (d, J = 5.6 Hz, 2H, ArH). ^{13}C-NMR (125 MHz, DMSO-d_6): δ 176.5, 158.5, 144.1, 143.3, 142.7, 136.6, 131.8, 130.6, 130.6, 128.9, 128.9, 121.9, 121.9, 120.2, 115.0, 93.8, 72.1; HREI-MS: m/z calcld for $C_{17}H_{11}ClN_4O_2S_2$ $[M]^+$ 402.0007 Found 402.0002.

3.6.16. methyl(Z)-4-((6-(2,3-dihydrobenzo[b][1,4]dioxin-2-yl)-3H-[1,3,4]thiadiazolo[2,3-c][1,2,4]thiadiazol-3-ylidene)amino)benzoate **(14)**

Yield: 70%; mp 216-217°C; ^{1}H-NMR (500 MHz, DMSO-d_6): δ 8.18 (d, J = 7.2 Hz, 2H, ArH), 7.83 (d, J = 6.8 Hz, 2H, ArH), 7.31 (d, J = 6.6 Hz, 2H, ArH),7.21 (d, J = 6.5 Hz, 2H, ArH), 5.52 (t, J = 5.7 Hz, 1H, ArH), 5.16 (d, J = 5.6 Hz, 2H, ArH), 3.87 (s, 3H, OCH3). ^{13}C-NMR (125 MHz, DMSO-d_6): δ 175.5, 165.9, 158.4, 144.1, 143.3, 142.7, 138.0, 132.4, 130.0, 130.0, 129.1,

129.1, 120.9, 120.9, 120.2, 115.0, 93.8, 66.1, 51.5; HREI-MS: m/z calcld for $C_{19}H_{14}N_4O_4S_2$ $[M]^+$ 426.0451 Found 426.0446.

3.6.17. (Z)-6-(2,3-dihydrobenzo[b][1,4]dioxin-2-yl)-N-(o-tolyl)-3H-[1,3,4]thiadiazolo[2,3-c][1,2,4]thiadiazol-3-imine **(15)**

Yield: 74%; mp 188-189°C; ^{1}H-NMR (500 MHz, DMSO-d_6): δ 7.70 (d, J = 7.1 Hz, 1H, ArH), 7.27 (m, 3H, ArH), 7.14 (d, J = 6.4 Hz, 2H, ArH), 7.03 (d, J = 6.4 Hz, 2H, ArH), 5.34 (t, J = 5.6 Hz, 1H, ArH), 5.03 (d, J = 5.6 Hz, 2H, ArH), 2.45 (s, 3H, CH3), 13CNMR (125 MHz, DMSO-d6) δ 176.5, 158.5, 146.3, 143.3, 142.7, 135.3, 131.1, 130.9, 129.0, 126.5, 125.8, 121.9, 121.9, 120.2, 115.0, 95.8, 65.1, 18.9; HREI-MS: m/z calcld for $C_{18}H_{14}N_4O_2$ S_2 $[M]^+$ 382.0553 Found 382.0548.

3.6.18. (Z)-6-(2,3-dihydrobenzo[b][1,4]dioxin-2-yl)-N-(p-tolyl)-3H-[1,3,4]thiadiazolo[2,3-c][1,2,4]thiadiazol-3-imine **(16)**

Yield: 71%; mp 187-188°C; ^{1}H-NMR (500 MHz, DMSO-d_6): δ 7.86 (d, J = 7.4 Hz, 1H, ArH), 7.36 (d, J = 6.8 Hz, 2H, ArH), 7.07 (d, J = 6.3 Hz, 2H, ArH), 7.02 (d, J = 6.4 Hz, 2H, ArH), 5.38 (t, J = 5.7 Hz, 1H, ArH), 5.06 (d, J = 5.4 Hz, 2H, ArH), 2.36 (s, 3H, CH3). ^{13}C-NMR (125 MHz, DMSO-d_6): δ 173.5, 158.7, 144.1, 143.3, 142.7, 140.7, 130.7, 128.1, 126.1, 126.1, 123.9, 122.9, 122.1, 120.2, 115.0, 93.8, 43.1, 21.3; HREI-MS: m/z calcld for $C_{18}H_{14}N_4O_2$ S_2 $[M]^+$ 382.0553 Found 382.0548.

3.6.19. (Z)-N-(3-chlorophenyl)-6-(2,3-dihydrobenzo[b][1,4]dioxin-2-yl)-3H-[1,3,4]thiadiazolo[2,3-c][1,2,4]thiadiazol-3-imine **(17)**

Yield: 73%; mp 203-204°C; ^{1}H-NMR (500 MHz, DMSO-d_6): δ 7.64 (s, 1H, ArH), 7.61 (d, J = 7.1 Hz, 1H, ArH), 7.51 (d, J = 6.8 Hz, 1H, ArH), 7.41 (t, J = 6.9 Hz, 1H, ArH), 7.23 (d, J = 6.4 Hz, 2H, ArH), 7.14 (d, J = 6.4 Hz, 2H, ArH), 5.45 (t, J = 5.6 Hz, 1H, ArH), 5.06 (d, J = 5.5 Hz, 2H, ArH). ^{13}C-NMR (125 MHz, DMSO-d_6): δ 175.5, 158.3, 146.8, 143.3, 141.7, 136.1, 134.4, 132.1, 130.2, 127.9, 125.3, 125.1, 124.9, 120.2, 116.0, 96.8, 43.1; HREI-MS: m/z calcld for $C_{17}H_{11}ClN_4O_2S_2$ $[M]^+$ 402.0007 Found 402.0002.

3.6.20. ((Z)-2-((6-(2,3-dihydrobenzo[b][1,4]dioxin-2-yl)-3H-[1,3,4]thiadiazolo[2,3-c][1,2,4]thiadiazol-3-ylidene)amino)phenol **(18)**

Yield: 67%; mp 190-191ºC; ^{1}H-NMR (500 MHz, DMSO-d_6): δ 10.66 (s, 1H, OH), 7.44 (d, J = 7.3 Hz, 1H, ArH), 7.31 (t, J = 7.1 Hz, 1H, ArH), 7.19 (d, J = 6.5 Hz, 2H, ArH), 7.10 (d, J = 6.4 Hz, 2H, ArH), 6.88 (d, J = 6.9 Hz, 1H, ArH), 6.85 (t, J = 6.9 Hz, 1H, ArH), 5.39 (t, J = 5.5 Hz, 1H, ArH), 5.13 (d, J = 5.3 Hz, 2H, ArH). ^{13}C-NMR (125 MHz, DMSO-d_6): δ 173.5, 158.2, 156.7, 146.0, 143.3, 142.7, 132.4, 126.5, 122.2, 121.9, 121.4, 120.9, 118.5, 117.8, 115.0, 93.8, 66.1; HREI-MS: m/z calcld for $C_{17}H_{12}N_4O_3S_2$ $[M]^+$ 384.0378 Found 384.0373.

3.6.21. (Z)-6-(2,3-dihydrobenzo[b][1,4]dioxin-2-yl)-N-(m-tolyl)-3H-[1,3,4]thiadiazolo[2,3-c][1,2,4]thiadiazol-3-imine **(19)**

Yield: 75%; mp 186-187 ºC; ^{1}H-NMR (500 MHz, DMSO-d_6): δ 7.71 (s, 1H, ArH), 7.63 (d, J = 7.1 Hz, 1H, ArH), 7.36 (t, J = 7.1 Hz, 1H, ArH), 7.22 (d, J = 6.7 Hz, 1H, ArH), 7.11 (d, J = 6.5 Hz, 2H, ArH), 7.04 (d, J = 6.5 Hz, 2H, ArH), 5.39 (t, J = 5.5 Hz, 1H, ArH), 5.04 (d, J = 5.3 Hz, 2H, ArH), 2.42 (s, 3H, CH3). ^{13}C-NMR (125 MHz, DMSO-d_6): δ 175.5, 158.5, 145.8, 143.3, 142.7, 136.5, 132.6, 131.3, 129.4, 128.7, 126.2, 120.9, 120.9, 120.2, 115.0, 93.8, 66.1, 21.3; HREI-MS: m/z calcld for $C_{18}H_{14}N_4O_2S_2$ $[M]^+$ 382.0553 Found 382.0548.

3.6.22. (Z)-5-((6-(2,3-dihydrobenzo[b][1,4]dioxin-2-yl)-3H-[1,3,4]thiadiazolo[2,3-c][1,2,4]thiadiazol-3-ylidene)amino)-2-methoxyphenol **(20)**

Yield: 71%; mp 212-213ºC; ^{1}H-NMR (500 MHz, DMSO-d_6): δ 9.33 (s, 1H, OH), 7.41 (s, 1H, ArH), 7.23 (d, J = 6.5 Hz, 2H, ArH), 7.17 (d, J = 6.3 Hz, 2H, ArH), 7.08 (d, J = 6.8 Hz, 1H, ArH), 6.92 (d, J = 6.9 Hz, 1H, ArH), 5.48 (t, J = 5.7 Hz, 1H, ArH), 5.16 (d, J = 5.6 Hz, 2H, ArH), 3.82 (s, 3H, OCH3). ^{13}C-NMR (125 MHz, DMSO-d_6): δ 175.5, 158.7, 152.4, 147.3, 146.8, 143.3, 142.7, 131.0, 122.8, 120.9, 120.9, 120.2, 115.9, 115.0, 112.3, 93.8, 66.8, 56.1; HREI-MS: m/z calcld for $C_{18}H_{14}N_4O_4S_2$ $[M]^+$ 414.0452 Found 414.0447.

3.6.23. (Z)-6-(2,3-dihydrobenzo[b][1,4]dioxin-2-yl)-N-(2,4-dimethoxyphenyl)-3H-[1,3,4]thiadiazolo[2,3-c][1,2,4]thiadiazol-3-imine **(21)**

Yield: 69%; mp 218-219ºC; ^{1}H-NMR (500 MHz, DMSO-d_6): δ 7.58 (d, J = 6.9 Hz, 1H, ArH), 7.14 (d, J = 6.3 Hz, 2H, ArH), 7.04 (d, J = 6.4 Hz, 2H, ArH), 6.69 (s, 1H, ArH), 6.64 (d, J = 6.6 Hz, 1H, ArH), 5.37 (t, J = 5.4 Hz, 1H, ArH), 5.08(d, J = 5.14 Hz, 2H, ArH), 3.84 (s, 3H, OCH3), 3.82 (s, 3H, OCH_3). ^{13}C-NMR (125 MHz, DMSO-d_6): δ 171.5, 163.9, 159.5, 158.5, 146.0, 145.4,

142.7, 133.0, 120.9, 120.9, 120.2, 115.0, 111.2, 106.7, 101.5, 90.8, 66.1, 55.8, 55.8; HREI-MS: m/z calcld for $C_{19}H_{16}N_4O_4S_2$ $[M]^+$ 428.0608 Found 428.0603.

3.6.24. (Z)-3-((6-(2,3-dihydrobenzo[b][1,4]dioxin-2-yl)-3H-[1,3,4]thiadiazolo[2,3-c][1,2,4]thiadiazol-3-ylidene)amino)benzene-1,2-diol **(22)**

Yield: 70%; mp 198-199°C; ^{1}H-NMR (500 MHz, DMSO-d_6): δ 10.94 (s, 1H, OH), 9.65 (s, 1H, OH), 7.22 (d, J = 6.9 Hz, 2H, ArH), 7.18 (d, J = 6.7 Hz, 2H,ArH), 6.97 (d, J = 6.4 Hz, 1H, ArH), 6.78 (d, J = 6.2 Hz, 1H, ArH), 6.70 (t, J = 6.3 Hz, 1H, ArH), 5.39 (t, J = 5.5 Hz, 1H, ArH), 5.14 (d, J = 5.4 Hz, 2H, ArH). ^{13}C-NMR (125 MHz, DMSO-d_6): δ 175.5, 159.5, 151.7, 146.9, 146.0, 143.3, 142.7, 124.7, 122.8, 120.9, 120.9, 120.2, 119.9, 119.6, 115.0, 93.8, 66.3; HREI-MS: m/z calcld for $C_{17}H_{12}N_4O_4S_2$ $[M]^+$ 400.0293 Found 400.0287.

3.6.25. ((Z)-6-(2,3-dihydrobenzo[b][1,4]dioxin-2-yl)-N-(2-fluorophenyl)-3H-[1,3,4]thiadiazolo[2,3-c][1,2,4]thiadiazol-3-imine **(23)**

Yield: 72%; mp 193-194°C; ^{1}H-NMR (500 MHz, DMSO-d_6): δ 7.74 (d, J = 7.4 Hz, 1H, ArH), 7.41 (t, J = 7.2 Hz, 1H, ArH), 7.33 (d, J = 7.1 Hz, 1H, ArH), 7.29 (d, J = 6.7 Hz, 2H, ArH), 7.25 (t, J = 7.1 Hz, 1H, ArH), 7.21 (d, J = 6.4 Hz, 2H, ArH), 5.49 (t, J = 5.8 Hz, 1H, ArH), 5.13 (d, J = 5.5 Hz, 2H, ArH). ^{13}C-NMR (125 MHz, DMSO-d_6): δ 176.6, 160.6, 159.2, 143.3, 142.7, 142.3, 132.6, 130.8, 124.4, 123.2, 122.9, 122.9, 120.2, 115.6, 115.0, 93.8, 66.1; HREI-MS: m/z calcld for $C_{17}H_{11}FN_4O_4S_2$ $[M]^+$ 386.0302 Found 386.0294.

3.6.26. ((Z)-6-(2,3-dihydrobenzo[b][1,4]dioxin-2-yl)-N-(3-fluorophenyl)-3H-[1,3,4]thiadiazolo[2,3-c][1,2,4]thiadiazol-3-imine **(24)**

Yield: 74%; mp 194-195 °C; ^{1}H-NMR (500 MHz, DMSO-d_6): δ 7.80 (s, 1H, ArH), 7.63 (t, J = 6.9 Hz, 1H, ArH), 7.55 (d, J = 6.9 Hz, 1H, ArH), 7.39 (d, J = 6.7 Hz, 1H, ArH), 7.33 (d, J = 6.5 Hz, 2H, ArH), 7.21 (d, J = 6.3 Hz, 2H, ArH), 5.51 (t, J = 5.7 Hz, 1H, ArH), 5.13 (d, J = 5.4 Hz, 2H, ArH). ^{13}C-NMR (125 MHz, DMSO-d_6): δ 175.5, 163.0, 158.5, 143.3, 142.7, 135.3, 130.4, 124.8, 120.9, 120.9, 120.2, 117.8, 115.0, 114.0, 93.8, 66.1, 146.8; HREI-MS: m/z calcld for $C_{17}H_{11}FN_4O_4S_2$ $[M]^+$ 386.0302 Found 386.0294.

3.6.27. ((Z)-6-(2,3-dihydrobenzo[b][1,4]dioxin-2-yl)-N-(4-fluorophenyl)-3H-[1,3,4]thiadiazolo[2,3-c][1,2,4]thiadiazol-3-imine **(25)**

Yield: 73%; mp 195-196°C; ^{1}H-NMR (500 MHz, DMSO-d_6): δ 7.79 (d, J = 7.1 Hz, 2H, ArH), 7.35 (d, J = 6.6 Hz, 2H, ArH),7.56 (d, J = 6.6 Hz, 2H, ArH), 7.28 (d, J = 6.5 Hz, 2H, ArH), 7.25 (d, J = 7.0 Hz, 2H, ArH), 5.51 (t, J = 5.7 Hz, 1H, ArH), 5.13 (d, J = 5.5 Hz, 2H, ArH). ^{13}C-NMR (125 MHz, DMSO-d_6): δ 174.5, 165.2, 158.3, 144.1, 143.3, 142.7, 130.8, 130.8, 129.3, 120.9, 120.9, 120.2, 115.6, 115.6, 115.0, 93.8, 66.1; HREI-MS: m/z calcld for $C_{17}H_{11}FN_4O_4S_2$ $[M]^+$ 386.0302 Found 386.0294.
